# Comparison of Airway Pressure Release Ventilation to High-Frequency Oscillatory Ventilation in Neonates with Refractory Respiratory Failure

**DOI:** 10.1155/2022/7864280

**Published:** 2022-05-02

**Authors:** Shreyas Arya, Melissa L. Kingma, Stacey Dornette, Amy Weber, Cathy Bardua, Sarah Mierke, Paul S. Kingma

**Affiliations:** ^1^Department of Neonatal/Perinatal Medicine, Wright State University Boonshoft School of Medicine, Dayton, Ohio, USA; ^2^The Perinatal Institute, Cincinnati Children's Hospital Medical Center, Cincinnati, Ohio, USA; ^3^Department of Pediatrics, University of Cincinnati College of Medicine, Cincinnati, Ohio, USA

## Abstract

**Background:**

Airway pressure release ventilation (APRV) is a relatively new mode of ventilation in neonates. We hypothesize that APRV is an effective rescue mode in infants failing conventional ventilation and it is comparable in survival rates to rescue with high-frequency oscillatory ventilation (HFOV).

**Methods:**

This is a 6-year retrospective cohort study of infants that failed synchronized intermittent mandatory ventilation (SIMV) and were rescued with either APRV or HFOV. For comparison, we divided infants into two groups (28-37 and >37 weeks) based on their corrected gestational age (CGA) at failure of SIMV.

**Results:**

Ninety infants were included in the study. Infants rescued with APRV (*n* = 46) had similar survival rates to those rescued with HFOV (*n* = 44)—28-37 weeks CGA (APRV 78% vs. HFOV 84%, *p* = 0.68) and >37 weeks CGA (APRV 76% vs. HFOV 72%, *p* = 0.74). Use of APRV was not associated with an increase in pneumothorax (APRV 0% and HFOV 10%, *p* = 0.31, in 28-37 weeks CGA, and APRV 0% and HFOV 4%, *p* = 0.22, in >37 weeks CGA).

**Conclusion:**

APRV can be effectively used to rescue infants with refractory respiratory failure on SIMV. When compared to HFOV, rescue with APRV is not associated with an increase in mortality or pneumothorax.

## 1. Introduction

In recent decades, despite progressive advances in survival of premature infants [1, 2], rates of bronchopulmonary dysplasia (BPD) have remained relatively constant [3]. Mechanical ventilation and oxygen therapy have long been recognized as mediators of lung injury. Philip described “oxygen plus pressure plus time” as the etiology of BPD in 1975 [4]. Since then, our understanding of ventilator-associated lung injury has improved, but the optimal lung-protective ventilation strategy remains elusive [5].

Concerns for lung injury from excessive pressures, hyperinflation, and atelectasis have led to the advent of alternative modes of ventilation. APRV was first described by Stock et al. [6] and is a form of pressure-limited ventilation with a time-cycled release phase [7]. The patient alternates between two pressures—“P-high” and “P-low” (release phase) for a time of “T-high” and “T-low,” respectively—but can continue to breathe spontaneously throughout all phases of the ventilator cycle [7].

APRV is used in adults with acute lung injury/acute respiratory distress syndrome (ALI/ARDS), and early application of APRV in adult trauma patients with ALI has been shown to have a tenfold reduction in ARDS and a threefold reduction in in-hospital mortality [8]. However, use of APRV in pediatric and neonatal patients is limited, and there is a paucity of human data regarding its use in this population. Most studies are limited to case reports and series or evaluation of a small number of patients [9, 10].

Use of APRV was initiated in the neonatal intensive care unit at our institution in 2008, and it has since become a frequently utilized mode of rescue ventilation, in addition to HFOV. We hypothesize that APRV can be effectively used as a rescue mode of ventilation in infants failing conventional ventilation and survival rates for these infants are comparable to those rescued with HFOV. To test this hypothesis, we evaluated retrospective outcome data in infants rescued with either APRV or HFOV.

## 2. Methods

After institutional review board approval, medical records of all infants that failed SIMV from January 2010 to December 2015 were reviewed. Our inclusion criteria consisted of infants of gestational age (GA) 24-42 weeks, >28 weeks corrected at failure of SIMV, and <1 year of chronological age, with need for rescue ventilation. Our exclusion criteria were need for rescue ventilation (APRV or HFOV) for less than 6 hours or its use with extracorporeal membrane oxygenation (ECMO). Infants with congenital diaphragmatic hernia, diffuse developmental disorders of the lung, or congenital surfactant deficiencies were also excluded.

### 2.1. Ventilator Characteristics

SIMV and APRV were delivered using Servo-i ventilators (MAQUET Medical Systems, USA) and HFOV using a SensorMedics 3100A ventilator (SensorMedics Corporation, USA). Assessment of failure of SIMV and choice of rescue ventilation was ultimately determined by the clinical judgement of the attending neonatologist. However, the typical criteria at our institution is a pCO_2_ > 65 mmHg and/or a FiO_2_ > 60%, despite peak ventilator pressures > 25 cmH_2_O. In general, when a patient failing SIMV was switched to APRV, they were started on a P-high that achieved appropriate chest rise and a T-high that achieved the desired respiratory rate. P-low was set at 0-3 cmH_2_O and T-low was set at 0.2-0.3 seconds to generate the desired measured positive end-expiratory pressure (PEEP). For HFOV, patients were started on a mean airway pressure (M_Paw_) that was 2-3 cmH_2_O greater than M_Paw_ on SIMV. Initial power was set to generate appropriate chest wiggle, and frequency was chosen based on CGA (term and postterm: 8 Hz; preterm: 10-12 Hz). Ventilator settings were titrated based on clinical judgement and blood gas measurements.

### 2.2. Clinical and Laboratory Data

Our primary outcome variable was survival, while our secondary outcome variable was the improvement in refractory respiratory failure, using APRV and HFOV rescue modes of ventilation. Patients were on continuous vital sign monitoring for the duration of mechanical ventilation. Blood gases were drawn to assess clinical status, based on the discretion of the attending neonatologist. Information collected from medical records included patient demographics (GA, birth weight, and sex), primary and secondary diagnoses, age and weight at failure of SIMV, length of hospital stay, survival to discharge, and incidence of pneumothorax. To evaluate the clinical status prior to rescue, blood gas and vital signs were recorded from measurements obtained immediately prior to escalation to rescue mode. To evaluate the clinical status following stabilization on the rescue mode, ventilator settings, blood gas, and vital signs were recorded at the time of the second blood gas measurement following rescue. Evaluation at the time of the second blood gas following rescue permitted the clinicians to make one set of ventilator adjustments with the first blood gas and then allowed the patient to adjust to those changes prior to the second blood gas.

### 2.3. Statistical Analysis

For comparison of APRV to HFOV, infants were divided into two groups based on CGA at failure of SIMV: 28-37 weeks (preterm) and >37 weeks (term). A paired two-tailed Student's *t*-test was used to compare continuous variables. Chi square was used for categorical variables. Statistical significance was defined as *p* value < 0.05. Results were expressed as the mean with standard deviations or median with interquartile range for continuous variables or as a percentage (%) for categorical variables.

Yehya et al. [11] compared APRV to HFOV in pediatric respiratory failure. The average incidence of mortality in their study was 39%. Assuming this as the baseline incidence and assuming *α* error of 5% and power of 80%, the sample size calculated would be 168 (84 per group) to detect a 50% change in mortality [11].

## 3. Results

During the 6-year study period, 112 infants met the inclusion criteria and required escalation to either HFOV or APRV, following refractory respiratory failure on SIMV. Thirteen infants in the APRV group and nine infants in the HFOV group were on the given mode of ventilation for less than 6 hours and were therefore removed from the data set leaving 90 total infants for further analysis (HFOV *n* = 44, APRV *n* = 46). All of the infants were delivered outside of our institution, none of the infants received surfactant while on APRV or HFOV, and none of the infants were escalated to ECMO. Three infants in the HFOV group and four infants in the APRV group had hemodynamically significant patent ductus arteriosus on echocardiogram. Two infants in the APRV group and three infants in the HFOV group received paralytics during the rescue period. Beyond the common diagnosis of prematurity (HFOV *n* = 19, APRV *n* = 9), patent ductus arteriosus (HFOV *n* = 3, APRV *n* = 4), and meconium aspiration syndrome (HFOV *n* = 4, APRV *n* = 3), infants were also diagnosed with necrotizing enterocolitis, gastroschisis, subglottic stenosis, tracheomalacia, congenital pulmonary airway malformation, vein of Galen malformation, tracheoesophageal fistula, hypoxic ischemic encephalopathy, and cystic teratoma.

### 3.1. Infants Failing SIMV and Rescued with APRV

**Figure 1:**
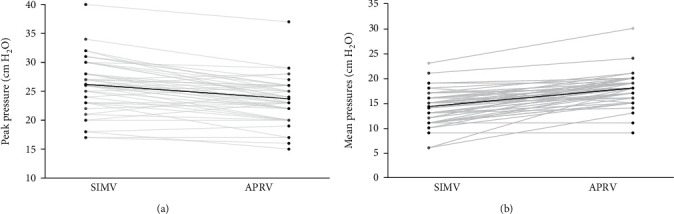
(a) Decrease in peak pressures with use of airway pressure release ventilation (APRV) for rescue, following failure on synchronized intermittent mandatory ventilation (SIMV). (b) Associated increase in mean airway pressures with APRV as compared to SIMV. Closed circles to the left represent SIMV and to the right represent APRV. Transparent gray lines represent individual patients. Solid black line represents mean.

Infants rescued with APRV saw significant reductions in peak pressures (SIMV 26.2 ± 4.5 vs. APRV 23.7 ± 3.7 cmH_2_O, *p* value < 0.001) (Figure 1(a)) and measured PEEP (SIMV 7.4 ± 1.9 vs. APRV 6.3 ± 1.9 cmH_2_O, *p* value < 0.001), following rescue, as compared to SIMV at failure, but the M_Paw_ was significantly higher on APRV (SIMV 14.3 ± 3.2 vs. APRV 18 ± 3.2 cmH_2_O, *p* value < 0.001) (Figure 1(b)). These infants also had a significant improvement in ventilation (SIMV pCO_2_78.9 ± 24.6 vs. APRV pCO_2_59 ± 10.1 mmHg, *p* value < 0.001) and a trend towards improved oxygenation (Table 1).

### 3.2. Comparison of APRV to HFOV 28-37 Weeks Corrected

Infants rescued with APRV had a median GA of 31.3 weeks and were corrected to 40.4 weeks with a mean weight of 3138 ± 1057 grams at rescue, while those of the HFOV group had a median GA of 34.1 weeks and were 37.6 weeks corrected with a mean weight of 2408 ± 834 grams at rescue. To account for the differences in GA and weight, further comparisons were made after matching infants for CGA at failure. After matching, there were no differences between the two groups in terms of GA, CGA, and weight at failure for the 28-37-week infants (Table 2).

For these infants, HFOV was employed for rescue at significantly lower M_Paw_ on SIMV, as compared to APRV (SIMV M_Paw_ at failure: APRV 15 ± 2.4 vs. HFOV 12.2 ± 1.5 cmH_2_O, *p* value 0.01). There was a difference in mean arterial blood pressure (MAP) between the two groups (APRV 47.8 ± 10.9 vs. HFOV 36.6 ± 11.1 mmHg, *p* value 0.02), but the relative change in MAP was not clinically significant, after the use of either mode (APRV 42.4 ± 7.8 vs. HFOV 34.4 ± 9.4 mmHg, *p* value 0.03). There was also a significant difference in pH between the APRV and HFOV groups (7.35 ± 0.1 vs. 7.26 ± 0.1, *p* value 0.05) due to the higher degree of metabolic acidosis in the HFOV group prior to rescue (base excess APRV 6.6 ± 5.7 vs. HFOV −1.3 ± 6.4, *p* value 0.005). There was no difference in survival between APRV (78%) and HFOV (84%) (*p* value 0.68). Despite higher M_Paw_ at rescue (APRV 17.6 ± 2.0 vs. HFOV 14.5 ± 4.6 cmH_2_O, *p* value 0.02), there were no pneumothoraces on APRV. In contrast, 10% of HFOV infants developed a pneumothorax; the difference was not statistically significant. APRV achieved similar oxygenation and ventilation as compared to HFOV.

### 3.3. Comparison of APRV to HFOV > 37 Weeks Corrected

For the >37-week group, APRV infants were significantly younger in median GA at birth (APRV 33.6 vs. HFOV 37.6, *p* value 0.04) but older in median CGA at failure (APRV 40.9 vs. HFOV 39.0 weeks, *p* value < 0.001) (Table 3). However, there was no difference in CGA at discharge. The two groups were also similar in SIMV settings at failure. The HFOV infants were significantly more acidotic at failure (APRV 7.30 ± 0.1 vs. HFOV 7.20 ± 0.1, *p* value 0.02) and remained so at the time of the second blood gas following rescue (APRV 7.36 ± 0.1 vs. HFOV 7.26 ± 0.1, *p* value < 0.001). There was no difference in survival between APRV (76%) and HFOV (72%) (*p* value 0.74). There were no pneumothoraces with APRV, whereas one infant developed a pneumothorax with HFOV; the difference was not statistically significant. APRV achieved similar oxygenation and ventilation as compared to HFOV.

Our comparisons suggest that survival rates are similar in infants rescued with APRV when compared to HFOV. To determine if there were any specific ventilatory parameters that were associated with a greater chance of survival, we compared infants that survived to those that did not survive, on each mode of ventilation (Table 4). In the APRV group, lower FiO_2_ (survivor 63 ± 25 vs. nonsurvivor 82 ± 26, *p* = 0.03), P-low (survivor 2.9 ± 1.1 vs. nonsurvivor 4.1 ± 2.0 cmH_2_O, *p* = 0.02), and measured PEEP (survivor 5.8 ± 1.5 vs. nonsurvivor 7.4 ± 2.3 cmH_2_O, *p* = 0.01) were associated with a higher rate of survival, while in the HFOV group, a lower M_Paw_ (survivor 15.7 ± 3.8 vs. nonsurvivor 18.7 ± 4.8 cmH_2_O, *p* = 0.04) was associated with a higher rate of survival.

## 4. Discussion

Our study represents a single center experience with the use of APRV for rescue ventilation, using a cohort of HFOV infants as a reference group for comparison. This is the largest cohort of infants rescued with APRV reported in the literature. APRV was comparable to HFOV in survival rates of both term and preterm infants with refractory respiratory failure on SIMV. In addition, the use of APRV was associated with an improvement in ventilation in neonates failing conventional ventilation and showed a trend towards improved oxygenation.

APRV used peak pressures that were ~3 cmH_2_O lower to achieve a M_Paw_ that was ~4 cmH_2_O higher than on SIMV. APRV achieved this higher M_Paw_ by maintaining peak pressures (P-high) for a prolonged period of time (T-high). These findings are consistent with results of other adult [12] and pediatric APRV studies [10]. Yehya et al. compared APRV to HFOV in older pediatric patients with hypoxemic respiratory failure on conventional ventilation and similarly found that APRV was not associated with increased mortality [11].

There was a substantial decrease in pCO_2_ following rescue with APRV. While the ventilator rate on APRV was ~6 breaths per minute higher than on SIMV, a change of this magnitude would not usually result in the ~20 mmHg reduction in pCO_2_ that we observed. The reasons for improved ventilation are unclear, but it is possible that the prolonged P-high phase of APRV reduces airway collapse at end-expiration, thereby promoting uninterrupted airflow. In addition, an infant on APRV can continue to breathe spontaneously throughout all phases of the ventilator cycle, which likely contributed to the improvement in ventilation.

In our current clinical practice, pressure-controlled SIMV is used as a primary mode and APRV and HFOV are used as rescue modes. APRV and HFOV may occasionally be used as primary modes for infants that may benefit from limiting peak pressures. For APRV, we start infants on a P-high 2-4 cmH_2_O below peak inspiratory pressure (PIP) on SIMV and have found that this usually achieves appropriate chest rise. Following initiation, P-high is titrated to achieve appropriate M_Paw_ and oxygenation goals. T-high is changed to affect ventilator rate. P-low and T-low are kept within the range of 0-3 cmH_2_O and 0.2-0.3 seconds, respectively. This short release assists in unloading CO_2_ from the airways and augments ventilation [7]. While Habashi originally suggested setting T-low to end at 75% of peak expiratory flow rates [12], the short expiratory times in neonates make this approach difficult. Therefore, our practice prefers to adjust P-low and T-low to achieve the patient-specific desired measured PEEP. However, caution must be used when using this strategy, since measured PEEP on the ventilator is not always precise and can change breath-to-breath with changes in the resistance of the ventilator circuit.

While the significant heterogeneity in the etiology of respiratory failure makes direct comparisons between APRV and HFOV difficult, our results suggest that APRV may be comparable to HFOV in achieving oxygenation and ventilation goals, when used for rescue. There was a substantial decrease in pCO_2_ after initiation of both rescue modes. Despite significantly higher M_Paw_ with APRV as compared to SIMV, there were no clinically significant changes in vital signs and no pneumothorax. In contrast, three infants rescued with HFOV developed a pneumothorax after its initiation. In the past, there have been concerns of overdistension, lung injury, and air leaks with prolonged inspiratory times [13, 14], but a large review by Jain et al. suggests that APRV is associated with no significant adverse outcomes [12] and recent animal studies indicate that APRV may be lung-protective [15, 16]. Clearly, there are multiple factors beyond ventilation that can contribute to infant mortality, and it is extremely difficult to control for those variables in a retrospective analysis of this size. We performed an initial analysis to see if there were variables associated with survival in our cohort. As anticipated, we observed a significant association between survival and lower M_Paw_ in the HFOV group; however, there was no association between survival and lower peak pressures or M_Paw_ in the APRV group. Interestingly, the most significant association in the APRV group was lower measured PEEP and P-low in the infants that survived.

HFOV was first used in neonates in the 1980s and has since become the preferred mode of rescue ventilation [17]. Similar to HFOV, an “open lung ventilation” approach is central to the success of APRV. Sustained inspiratory pressures allow the patient to maintain an open and stabilized lung which allows breathing to occur from a more compliant portion of the pressure-volume curve [7]. However, a major potential advantage of APRV over HFOV is its favorable hemodynamic profile. Walsh et al. found that unrestricted spontaneous breathing during APRV causes intermittent negative intrathoracic pressures restoring normal cardiopulmonary interaction leading to improved cardiac output and pulmonary perfusion [9]. In adults, APRV has been associated with improved renal perfusion [18], and animal studies have shown improved splanchnic perfusion with APRV, as compared to conventional ventilation [19]. These benefits might be critical to using APRV in neonates.

One of the limitations of our study is the retrospective design. Also, since clinical judgement was used to assess failure of SIMV and choose the rescue mode, there is a possibility of bias for when to declare failure and which rescue mode to choose. We attempted to reduce some bias by matching infants by CGA at failure of SIMV. Dividing infants into preterm and term for comparison led to a smaller sample size, but these subgroups were similar for the majority of the studied parameters prior to rescue. However, since this was not a prospective randomized trial, some significant differences between the APRV and HFOV groups existed, limiting the reliability of comparisons. We excluded infants < 28 − week CGA because there were not enough of these infants rescued with APRV to make meaningful comparisons. It is also important to note that we compared two pressure modes of ventilation. Growing evidence suggests that volume modes of ventilation may be preferable for infants; therefore, APRV may have limited benefit in infants failing volume modes.

A recent prospective trial compared APRV to HFOV in pediatric patients with ARDS. This study of 52 patients was terminated early when a trend towards higher mortality was observed in the APRV arm. Our study of 90 infants did not suggest a higher mortality in APRV patients. There is no clear explanation for the conflicting results. However, several factors in the Lalgudi Ganesan study suggest that the APRV group was younger and sicker than the HFOV group at the time of randomization [20].

In conclusion, our data suggests that APRV is an effective rescue mode of ventilation in infants failing SIMV. For these infants, it is also comparable in survival rates to HFOV and can achieve similar ventilation and oxygenation goals. APRV can serve as an important tool in the armamentarium of providers managing neonatal respiratory failure. However, further prospective randomized trials are needed to better define its safety and efficacy and to compare it to the current benchmarks of neonatal ventilation.

## Figures and Tables

**Table 1:** Infants failing SIMV rescued with APRV.

	SIMV at failure (*n* = 46)	APRV at rescue (*n* = 46)	*p* value
Peak pressures (cmH_2_O)	26.2 ± 4.5	23.7 ± 3.7	<0.001
Measured PEEP (cmH_2_O)	7.4 ± 1.9	6.3 ± 1.9	<0.001
Ventilator rate	45.7 ± 11.6	51.9 ± 10.3	0.001
Ventilator M_Paw_ (cmH_2_O)	14.3 ± 3.2	18.0 ± 3.2	<0.001
FiO_2_ (%)	73.0 ± 27	69.5 ± 27	0.13
pH^†^	7.30 ± 0.12	7.36 ± 0.12	<0.001
pCO_2_^†^ (mmHg)	78.9 ± 24.6	59.0 ± 10.1	<0.001
Base excess^†^	7.4 ± 8.0	7.0 ± 8.4	0.55

^†^Blood gas values for SIMV at the time of failure and for APRV at the time of the second blood gas after rescue. PEEP: positive end-expiratory pressure; M_Paw_: mean airway pressure; FiO_2_: fractional inspired oxygen; pCO_2_: partial pressure of carbon dioxide. Data expressed as mean ± standard deviation.

**Table 2:** Infants grouped by corrected gestational age (CGA) at failure (28-37 weeks).

		APRV (*n* = 9) rescue	HFOV (*n* = 19) rescue	*p* value
Patient characteristics	GA at birth (wk)^∗^	26.7 (25.3-28.0)	28.9 (27.8-30.3)	0.24
	CGA at failure (wk)^∗^	33.4 (32.3-34.1)	32.7 (29.8-34.3)	0.20
	Birth weight (g)	1170 ± 785	1376 ± 730	0.51
	Weight at failure (g)	1843 ± 553	1693 ± 707	0.58
	CGA at discharge (wk)^∗^	44.0 (32.4-46.6)	39.9 (38.6-41.8)	0.99
	Pneumothorax *n* (%)	0 (0)	2 (10)	0.31
	Survival *n* (%)	7 (78)	16 (84)	0.68

Patient status at failure of SIMV	PIP (cmH_2_O)	26.4 ± 3.6	22.5 ± 3.3	0.01
	Measured PEEP (cmH_2_O)	7.4 ± 1.9	5.6 ± 0.8	0.02
	Ventilator rate	50.0 ± 8.3	47.4 ± 9.6	0.46
	Ventilator M_Paw_ (cmH_2_O)	15.0 ± 2.4	12.2 ± 1.5	0.01
	pH	7.2 ± 0.1	7.1 ± 0.1	0.03
	pCO_2_ (mmHg)	81.7 ± 31.3	91.0 ± 22.8	0.44
	Base excess	6.6 ± 5.7	−1.3 ± 6.4	0.005
	FiO_2_ (%)	74.0 ± 31.5	72.4 ± 30.4	0.89
	Heart rate	156 ± 14	149 ± 20	0.28
	Respiratory rate	50.7 ± 8.8	49.2 ± 17.0	0.76
	BP MAP (mmHg)	47.8 ± 10.9	36.6 ± 11.1	0.02

Patient status at rescue^†^	Ventilator M_Paw_ (cmH_2_O)	17.6 ± 2.0	14.5 ± 4.6	0.02
	pH	7.35 ± 0.1	7.26 ± 0.1	0.05
	pCO_2_ (mmHg)	54.9 ± 14.3	60.0 ± 19.0	0.44
	Base excess	4.4 ± 7.6	−1.3 ± 5.6	0.06
	FiO_2_ (%)	64.2 ± 27.8	62.2 ± 31.7	0.86
	Heart rate	153 ± 20	148 ± 19	0.53
	BP MAP (mmHg)	42.4 ± 7.8	34.4 ± 9.4	0.03

^†^Values for both APRV and HFOV at the time of the second blood gas after rescue. PIP: peak inspiratory pressure; PEEP: positive end-expiratory pressure; pCO_2_: partial pressure of carbon dioxide, FiO_2_: fractional inspired oxygen, BP MAP: blood pressure mean arterial pressure; M_Paw_: mean airway pressure. Data expressed as median with interquartile range (∗) or mean ± standard deviation.

**Table 3:** Infants grouped by corrected gestational age (CGA) at failure (>37 weeks).

		APRV (*n* = 37) rescue	HFOV (*n* = 25) rescue	*p* value
Patient characteristics	GA at birth (wk)^∗^	33.6 (27.0-38.0)	37.6 (34.1-38.9)	0.04
	CGA at failure (wk)^∗^	40.9 (39.9-43.6)	39.0 (38.1-40.1)	<0.001
	Birth weight (g)	1860 ± 1142	2433 ± 1049	0.05
	Weight at failure (g)	3452 ± 900	2863 ± 540	0.004
	CGA at discharge (wk)^∗^	45.7 (39.4-50.0)	43.1 (41.7-45.8)	0.85
	Pneumothorax *n* (%)	0 (0)	1 (4)	0.22
	Survival *n* (%)	28 (76)	18 (72)	0.74

Patient status at failure of SIMV	PIP (cmH_2_O)	26.2 ± 4.7	26.1 ± 4.5	0.92
	Measured PEEP (cmH_2_O)	7.4 ± 2.0	6.6 ± 2.7	0.24
	Ventilator rate	44.6 ± 12.1	44.6 ± 12.6	0.99
	Ventilator M_Paw_ (cmH_2_O)	14.1 ± 3.4	13.8 ± 4.0	0.74
	pH	7.3 ± 0.1	7.2 ± 0.1	0.02
	pCO_2_ (mmHg)	78.2 ± 23.1	76.5 ± 26.5	0.79
	Base excess	7.6 ± 8.5	−0.1 ± 7.9	<0.001
	FiO_2_ (%)	72.8 ± 27.1	77.7 ± 24.1	0.45
	Heart rate	146 ± 25	144 ± 22	0.76
	Respiratory rate	49.5 ± 12.2	43.1 ± 24.1	0.22
	BP MAP (mmHg)	54.6 ± 11.6	48.2 ± 15.6	0.08

Patient status at rescue^†^	Ventilator M_Paw_ (cmH_2_O)	18.1 ± 3.4	17.8 ± 3.6	0.71
	pH	7.36 ± 0.1	7.26 ± 0.1	<0.001
	pCO_2_ (mmHg)	59.9 ± 19.5	60.4 ± 18.4	0.92
	Base excess	7.7 ± 8.5	0 ± 7.6	<0.001
	FiO_2_ (%)	71.0 ± 26.6	79.3 ± 25.9	0.22
	Heart rate	146 ± 26	141 ± 29	0.47
	BP MAP (mmHg)	49.7 ± 8.3	44.2 ± 11.8	0.048

^†^Values for both APRV and HFOV at the time of the second blood gas after rescue. PIP: peak inspiratory pressure; PEEP: positive end-expiratory pressure; pCO_2_: partial pressure of carbon dioxide; FiO_2_: fractional inspired oxygen; BP MAP: blood pressure mean arterial pressure; M_Paw_: mean airway pressure. Data expressed as median with interquartile range (∗) or mean ± standard deviation.

**Table 4:** Comparison of survivors versus nonsurvivors in each ventilator group.

	APRV			HFOV		
	Survivor (*n* = 34)	Nonsurvivor (*n* = 12)	*p* value	Survivor (*n* = 34)	Nonsurvivor (*n* = 10)	*p* value
pH	7.34 ± 0.1	7.39 ± 0.1	0.24	7.26 ± 0.1	7.25 ± 0.1	0.74
pCO_2_ (mmHg)	61 ± 19	54 ± 14	0.28	61 ± 17	58 ± 21	0.69
FiO_2_ (%)	63 ± 25	82 ± 26	0.03	74 ± 28	63 ± 32	0.29
P-high (cmH_2_O)	23.4 ± 2.8	23.7 ± 5.6	0.81			
P-low (cmH_2_O)	2.9 ± 1.1	4.1 ± 2.0	0.02			
Measured PEEP (cmH_2_O)	5.8 ± 1.5	7.4 ± 2.3	0.01			
T-high (s)	0.9 ± 0.4	0.8 ± 0.2	0.51			
T-low (s)	0.4 ± 0.1	0.3 ± 0.1	0.32			
Rate	51 ± 11	55 ± 8	0.29			
M_Paw_ (cmH_2_O)	17.6 ± 2.4	18.5 ± 4.5	0.42	15.7 ± 3.8	18.7 ± 4.8	0.04
Power				2.7 ± 0.8	2.5 ± 0.4	0.71
Amplitude				31.2 ± 6.5	35.0 ± 8.4	0.16
Frequency (Hz)				9.3 ± 1.1	9.6 ± 1.2	0.42
						

Values for both APRV and HFOV at the time of the second blood gas after rescue. pCO_2_: partial pressure of carbon dioxide; FiO_2_: fractional inspired oxygen; P-high: pressure high; P-low: pressure low; PEEP: positive end-expiratory pressure; T-high: time high; T-low: time low; M_Paw_: mean airway pressure. Data expressed as mean ± standard deviation.

## Data Availability

The data used to support the findings of this study are available from the corresponding author upon request.

## Abbreviations

APRV: Airway pressure release ventilation
M_Paw_: Mean airway pressure
HFOV: High-frequency oscillatory ventilation
SIMV: Synchronized intermittent mandatory ventilation
CGA: Corrected gestational age
BPD: Bronchopulmonary dysplasia
CPAP: Continuous positive airway pressure
CO_2_: Carbon dioxide
ALI: Acute lung injury
ARDS: Acute respiratory distress syndrome
GA: Gestational age
ECMO: Extracorporeal membrane oxygenation
PEEP: Positive end-expiratory pressure
pCO_2_: Partial pressure of carbon dioxide
PIP: Peak inspiratory pressure.

## Supplementary Materials

Supplementary Materials This document is a guideline for the use of Airway Pressure Release Ventilation (APRV) in Neonates which describes how this mode works, initial settings and how to make adjustments. These guidelines are are meant to serve as an initial introduction to APRV and the reader should not assume that these guidelines are sufficient education to begin utilizing APRV without additional training and supervision.
