# Supplementary material for: Comparison of Airway Pressure Release Ventilation to High-Frequency Oscillatory Ventilation in Neonates with Refractory Respiratory Failure
Source: Int J Pediatr. 2022 May 2;2022:7864280. doi: 10.1155/2022/7864280 (PMC9085362; doi:10.1155/2022/7864280)
Supplement: Supplementary Materials — This document is a guideline for the use of Airway Pressure Release Ventilation (APRV) in Neonates which describes how this mode works, initial settings and how to make adjustments. These guidelines are are meant to serve as an initial introduction to APRV and the reader should not assume that these guidelines are sufficient education to begin utilizing APRV without additional training and supervision. [file 7864280.f1.docx]

**Airway Pressure Release Ventilation in the Neonatal Intensive Care Unit: a guideline for use in the neonate.**

Melissa Kingma CNP; Paul Kingma MD PhD

Cincinnati Children's Hospital Medical Center, Cincinnati, Ohio, United States

Airway Pressure Release Ventilation (APRV, aka Bivent and Bilevel) is a relatively new mode of ventilation in the neonate that promotes an “open lung” ventilation strategy by using prolonged inspiratory times with periodic pressure releases. Although this creates an inverse inspiratory:expiratory ratio, which historically was associated with an increased risk of pneumothorax, this complication is theoretically reduced in APRV by allowing exhalation during all phases of the respiratory cycle. There are currently no clear trials supporting or refuting the use of APRV in the neonatal population, but the clinical experience at the author’s institution suggests that some infants respond very well to this mode of ventilation. As with all approaches to neonatal ventilation, the medical team should insure they are adequately trained in all of the components, display data, settings, risks and benefits associated with APRV before utilizing this mode of ventilation. The guidelines presented below are meant to serve as an initial introduction to APRV and in no way should the reader assume that these guidelines are sufficient education to begin utilizing APRV without additional training and supervision.

The critical settings in APRV are P-high, P-low, T-high, T-low and PS. The P-high is similar to peak inspiratory pressure (PIP) in pressure modes of ventilation but P-high required is usually 2-5 cm H_2_O lower than PIP. The P-low establishes the lower threshold of a pressure gradient which encourages the outflow of air during the exhalation phase, T-low. Ventilator rate is primarily set by lengthening or shortening the T-high and not T-low. The exhalation time, T-low, is adjusted to balance two competing purposes. The first is to provide adequate exhalation time to allow sufficient tidal volume, while the second is to stop exhalation before pulmonary pressures drop too low and atelectasis occurs. The adult and pediatric literature primarily describe two approaches to APRV. The first approach, commonly called the “Habashi method” sets the P-high to the desired plateau pressure used in conventional ventilation and the P-low to 0 cm H_2_O to maximize the pressure gradient and promote exhalation. The T-high is adjusted to the desired release rate and the T-low is set to provide the minimum time required to achieve adequate exhalation. The second approach is similar to prolonged inspiratory-time pressure-targeted ventilation. This approach sets the P-high and P-low to the expected upper and lower inflection points of the pressure volume curve (similar to PIP and PEEP on conventional ventilation). The T-low is lengthened to allow complete exhalation and minimal trapping of exhaled gas while the T-high is set to achieve an inverse T-high:T-low ratio of 1-3:1. Our experience with APRV in the NICU suggests that the unique respiratory mechanics of the newborn (*ie*. high respiratory rate and short expiratory time) make both of these approaches impractical and difficult to follow while maintaining patient stability. Therefore, we have developed an alternative approach at Cincinnati Children’s Hospital Medical Center that is described below.

The P-high is set to the desired plateau pressure of the infant to achieve adequate chest rise and oxygenation. For a typical newborn the P-high will be 13-25 cm H_2_O, but this will be highly dependent on the size of the infant, severity of respiratory failure and chest wall compliance. If an infant is transitioning from conventional pressure or volume modes, the P-high will usually be 2-5 cm H_2_O below the peak pressure utilized in the previous mode. The relative difference between the peak pressure and P-high is greater at higher peak pressures. For example, an infant that requires a PIP of 17 cm H_2_O will likely require a P-high of ~15 cm H_2_O while an infant that requires a PIP of 28 cm H_2_O will likely require a P-high of ~23 cm H_2_O.

The P-low is set to 0-5 cm H_2_O. Habashi suggests setting the P-low to 0 cm H_2_O, but our experience suggests that due to the short exhalation time of infants, a P-low of 0 cm H_2_O will lead to dangerously low auto PEEP and risk atelectasis even when utilizing a very short T-low. Therefore, we will typically start with a P-low of 3 cm H_2_O. This value may be decreased if a larger pressure gradient is required to encourage a larger tidal volume and more ventilation. Alternatively, the P-low may be increased if the auto PEEP measurements are low and the patient is at risk for atelectasis. It is important to note that the tidal volume and auto PEEP can also be influenced by the exhalation time permitted in the T-low setting.

The T-high is set to achieve the desired “release rate” or respiratory rate. The T-high in APRV is an absolute, not fractional, time value and is the primary control for the set respiratory rate. For example, when paired with a T-low of 0.2 seconds, a T-high of 0.8 seconds will result in a ventilator rate of 60 breaths per minute, while a T-high of 1.3 will equal a ventilator rate of 40 breaths per minute. It is possible to achieve a very high and well tolerated I:E ratio with APRV (*eg*. 1.6 second T-high to 0.2 second T-low equals a respiratory rate of 33 and a 8:1 I:E ratio).

The T-low is adjusted to maximize alveolar recruiting and tidal volumes. In general, a shorter T-low (*eg*. 0.2 seconds) will lead to higher PEEP at the end of the exhalation phase, less atelectasis and better alveolar recruiting. In contrast, a longer T-low will lead to prolonged exhalation, larger tidal volumes and lower CO_2_ levels. Usually, the ideal compromise for these competing processes is achieve with a T-low of 0.2-0.4 seconds. The Habashi method suggests adjusting the T-low so that exhalation stops when expiratory flow rates fall to 50-75% of the peak expiratory flow rate. Some ventilators will calculate this threshold for the user and adjust the T-low accordingly, but in general we have found this approach to be difficult to achieve in most patients. Alternatively, we adjust the T-low to achieve the desired auto PEEP in a given patient. If the auto PEEP is too low (*eg.* 2-3 cm H_2_O), the T-low will be decreased to stop exhalation before the pulmonary pressures drop below the desired PEEP levels (*eg.* 5-7 cm H_2_O). If the auto PEEP is too high, then the T-low will be increased to allow more exhalation and promote a lower auto PEEP. The time required to exhale will depend on multiple factors including the size of the infant, pulmonary compliance, tidal volume and the pressure gradient created by the P-high and P-low settings. Once an appropriate T-low is determined for an infant, it is important to resist the temptation to use the T-low to adjust the set respiratory rate for an infant.

Pressure support is set to 2-4 cm H_2_O in most infants. The pressure support may be increased as the APRV settings are weaned, and the infant has more spontaneous respirations.

When a patient improves and is ready for weaning from APRV, we will wean the P-high and T-high as appropriate in a given infant. If the patient is oxygenating well, but still has elevated CO_2_ levels then we will focus on weaning the P-high to decrease the mean airway pressure and resulting oxygenation. While weaning the P-high we will recognize that we also may be decreasing the tidal volume and therefore may further increase CO_2_ levels. Alternatively, if a patient is ventilating well with low CO_2_ levels, but still has poor oxygenation, then we will focus on weaning the respiratory rate by increasing the T-high. While increasing the T-high we will recognize that we also may be increasing the mean airway pressure by spending more time on P-high. If this occurs, we may need to decrease the P-high to maintain a constant mean airway pressure and resulting level of oxygenation. If a patient is both oxygenating well and has low CO_2_ levels, then we will wean the respiratory support by increasing the T-high and decreasing the P-high. Eventually the patient will be supported by the equivalent of “CPAP pressure support” ventilation. We have extubated several infants from APRV and found that conversion to a conventional mode of ventilation is not required for successful extubation.

Example patients:

Patient 1: Term infant, weight 3.0 kg, failing on conventional ventilation with peak inspiratory pressures of 25 cm H_2_O, PEEP 6 cm H_2_O, pressure support 10 cm H_2_O, ventilator rate of 50.

APRV settings: Approximate initial settings P-high 21 cm H_2_O, P-low 3 cm H_2_O, T-high 0.8 seconds, T-low 0.3 seconds (rate 54 breaths per minute), pressure support 2 cm H_2_O

Patient 2: 29-week infant, weight 0.9 kg, failing on conventional ventilation with peak inspiratory pressures of 20 cm H_2_O, PEEP 6 cm H_2_O, pressure support 6 cm H_2_O, ventilator rate of 60.

APRV settings: Approximate initial settings P-high 18 cm H_2_O, P-low 2 cm H_2_O, T-high 0.7 seconds, T-low 0.2 seconds (rate 67 breaths per minute), pressure support 2 cm H_2_O
